# The Correlation between Endotoxin, D-Lactate, and Diamine Oxidase with Endoscopic Activity in Inflammatory Bowel Disease

**DOI:** 10.1155/2022/9171436

**Published:** 2022-09-16

**Authors:** Qi Zhang, Xin Gao, Jixiong Wu, Min Chen

**Affiliations:** ^1^Department of General Medicine, Renmin Hospital of Wuhan University, Wuhan 430060, China; ^2^Department of Gastroenterology, Renmin Hospital of Wuhan University, Wuhan 430060, China; ^3^Department of Gastroenterology, Huanggang Central Hospital, Huanggang 438000, China; ^4^Department of Gastroenterology, Zhongnan Hospital of Wuhan University, Wuhan 430062, China

## Abstract

**Methods:**

A total of 149 eligible IBD patients including 82 Crohn disease (CD) and 67 Ulcerative colitis (UC) who had received both endoscopic examination and intestinal barrier function detection in our hospital were enrolled in this study. Endoscopic activity was estimated by the Simple Endoscopic Score (SES-CD) for Crohn's Disease and the ulcerative colitis endoscopic index of severity (UCEIS) for ulcerative colitis. The predictive value and optimal predictive thresholds for those biomarkers were determined by receiver operating characteristic analysis.

**Results:**

For UC patients, DAO, D-lactate, and ETX showed better correlation with UCEIS than erythrocyte sedimentation rate (ESR) and C-reactive protein (CRP) and exhibited satisfactory predictive value in predicting remission. Among patients with CD, DAO and ETX not only showed a better correlation than WBC, ESR, and CRP with SES-CD but also capable to identify more severe patients.

**Conclusion:**

DAO and ETX could be used to distinguish different endoscopic activity of CD. DAO, D-lactate, and ETX could predict UC endoscopic remission.

## 1. Introduction

The assessment of disease activity of inflammatory bowel disease (IBD) has crucial implications for making therapeutic strategies and predicting prognosis in clinical practice. Endoscopic remission in patients with IBD is associated with durable remission and is considered the ultimate goal of IBD therapy [1]. The gold standard of evaluating disease activity of IBD is endoscopic and histopathological examination. However, endoscopy examination with biopsy is an invasive, expensive, and time-consuming procedure. In clinical practice, some patients may be reluctant to handle fecal material. Therefore, it is important to find reliable serum biomarkers which can replace endoscopic examination to monitor disease activities or predict endoscopic remission.

The traditional biological activity markers of IBD that are widely used in clinical practice to evaluate disease activity of IBD are C-reactive protein (CRP) and erythrocyte sedimentation rate (ESR). However, both of them lack intestinal specificity. Some studies showed that the relationship between CRP and disease activity of Crohn disease (CD) was weak [2, 3]. Another study found that there was no relation between serum CRP and endoscopic activity of ulcerative colitis (UC), and ESR was the more accurate to assess the endoscopic activity than CRP with low sensitivity and specificity [4]. The effect of CRP and ESR in evaluating IBD is limited as previously described [5, 6]. An accurate and reliable noninvasive serum biomarker for evaluating the endoscopic activity of IBD has not yet been identified.

The barrier function of the intestine was found to play a significant role in IBD [7–9]. Previous studies have suggested that monitoring the change of intestinal barrier function may help evaluate disease status of IBD [10–12]. Diamine oxidase (DAO), D-lactate, and endotoxin (ETX) are three biomarkers which are most commonly used to assess the function of intestinal barrier [13–15]. Previous studies have identified that DAO, D-lactate, and ETX are relatable with IBD activity. Even so, whether these biomarkers were capable to replace the invasive endoscopic techniques was still unclear. In this study, we studied the correlation of DAO, D-lactate, and ETX levels with endoscopic activity in patients with IBD, aiming to assess whether DAO, D-lactate, and ETX could monitor the disease activity of IBD and predict the endoscopic remission.

## 2. Patients and Methods

### 2.1. Patients

A total of 149 IBD patients (82 CD, 67 UC) who have undergone both endoscopic examination and intestinal barrier function detection in the Department of Gastroenterology of Zhongnan Hospital of Wuhan University between December 2016 and October 2017 were included in this study. The diagnosis of IBD was made bases on a combined assessment of clinical features, endoscopic findings, histopathological findings, and results of follow-up evaluation, including response to medical therapy, such as corticosteroids, immunomodulators, and/or antitumor necrosis factor-*α* (TNF-*α*) biologics. All patients were informed and consented to the study. All research processes were carried out in accordance with relevant guidelines and ethics regulations and with approval from Zhongnan Hospital of Wuhan University, approval number: 2022104K.

### 2.2. Inclusion and Exclusion Criteria

The inclusion criteria were as follows: (1) diagnosed with IBD; (2) patients have undergone both endoscopic examination and intestinal barrier function detection; (3) the interval between endoscopic examination and intestinal barrier function was less than 3 weeks. The exclusion criteria were as follows: (1) patients who had previous surgery of bowel resection; (2) patients who had taken nonsteroidal anti-inflammatory drug (NSAID) in the previous 3 weeks; (3) patients with specific pathogens infection (such as HIV, hepatitis B, syphilis, and salmonella), renal failure, chronic liver, colorectal cancer, connective tissue diseases, or pancreatitis.

### 2.3. Protocol

Endoscopic disease activity was, respectively, scored by experienced endoscopist using the Simple Endoscopic Score for Crohn's Disease (SES-CD) or the ulcerative colitis endoscopic index of severity (UCEIS) [16, 17].

D-lactate, DAO, and ETX in the blood were colored by diamine oxidase, lactic acid, bacterial endotoxin assay kit (Enzyme), which was provided by Beijing Zhongsheng Jinyu Diagnostic Technology Ltd., China. Then, the stained samples were detected and quantified by JY-DLT Intestinal barrier function biochemical index analysis system.

### 2.4. Definitions of Variables

The “Big Ulcer” was defined as their longest diameter more than 20 mm, the “Small ulcers” were defined as whose longest diameter less than 5 mm. The calculation of SES-CD was based on four endoscopic variables (presence and size of ulcers, proportion of surface covered by ulcers, proportion of surface affected by disease, and presence and severity of stenosis) and assessed in 5 ileocolonic segments. Vascular pattern, bleeding, and erosions or ulcerations were evaluated in the most inflamed colonic segment to calculate the UCEIS. The SES − CD ≤ 2 or UCEIS ≤ 1 were defined as remission. For active CD, we defined SES-CD 3-6 as mild activity, 7-15 as moderate activity, and score ≥ 16 as severe activity. For active UC patients, we defined UCEIS 2-6 as mild and moderate activity and 7-8 as severe activity.

### 2.5. Statistical Analysis

Statistical analysis was performed using the SPSS 21.0 software (SPSS, Chicago, IL, USA) and GraphPad Prism 5.0 software (GraphPad Software Inc., La Jolla, CA, USA). Correlation between categorical variables were determined by Pearson correlation coefficient *r*. Receiver operating characteristic (ROC) analysis was used to find the optimal thresholds of the DAO, D-lactate, and ETX for discriminating endoscopic activity in patients with IBD. The optimal diagnostic thresholds for a test were determined by plotting Youden's index (*YI* = [sensitivity + specificity] − 1). The area under the curve (AUC) was used to compare accuracies of biomarkers in predicting different endoscopic severity. Sensitivity, specificity, positive predictive value (PPV), negative predictive value (NPV), and accuracy (proportion of correct diagnoses) were calculated to determine the reliability of each of the biomarkers to predict endoscopic severity in patients with IBD.

## 3. Results

### 3.1. Patient Baseline Characteristics

A total of 149 eligible patients (82 CD, 67 UC) were enrolled, 134 patients (73 CD, 61 UC) were in active, and 15 patients (9 CD, 6 UC) were in remission. The baseline characteristics of all patients were shown in Table 1. The median disease duration of all patients was 3 years (interquartile range [IQR]:1.0-4.0 yr). The patients with CD were younger than UC (median age 28.0 vs. 46.0 years, *P* < 0.001). There was no statistical difference in gender and smoking history between patients with UC and CD. Based on the Montreal classification, most of the patients with CD (55.65%) had an ileocolonic disease (L3), and 64.18% of the patients with UC had an extended colitis (E3).

### 3.2. Correlation between Biomarkers and Endoscopic Score in CD Patients

Correlation coefficient *r* between biomarkers and endoscopic score of disease severity of CD was shown in Table 2. As shown in the table, DAO and ETX were demonstrated to have a better correlation with SES-CD (*r* = 0.532 and *r* = 0.468, respectively; *P* < 0.0001 for both) compared to WBC, ESR, and CRP (*r* = 0.146, *P* = 0.191; *r* = 0.346, *P* = 0.0015; *r* = 0.250, *P* = 0.023), while there was no correlation between D-lactate and SES-CD (*r* = 0.167, *P* = 0.133).

### 3.3. Correlation between Biomarkers and Endoscopic Score in UC Patients

Correlation coefficient *r* between biomarkers and endoscopic score of disease severity of UC were shown in Table 3. Similar to CD, DAO and ETX were demonstrated to have better correlation with UCEIS (*r* = 0.600 and *r* = 0.505, respectively; *P* < 0.0001 for both) compared with WBC, ESR, and CRP (*r* = 0.151, *P* > 0.05; *r* = 0.285, *P* = 0.021; *r* = 0.334, *P* = 0.006). There was significant but weak correlation between D-lactate and UCEIS (*r* = 0.407, *P* = 0.0006). The relationship of DAO, D-lactate, and ETX with endoscopic score of UC were shown to be better than those of CD.

### 3.4. Value of Intestinal Barrier Marker in Predicting Endoscopic Severity in CD

**Figure 1:**
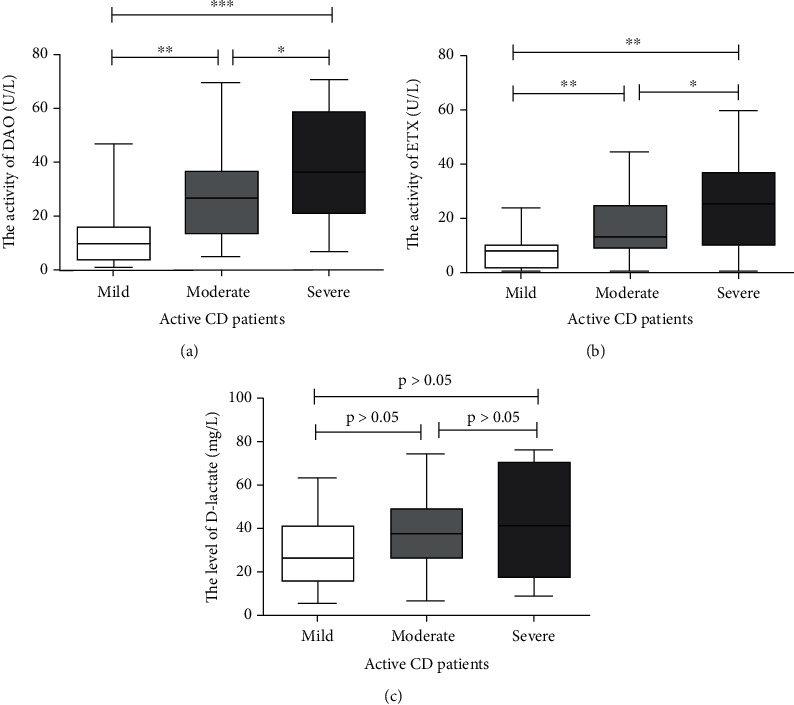
In active CD patients (SES − CD > 2), the DAO levels of severe groups were higher than moderate groups (*P* < 0.05, (a)), moderate groups were higher than mild groups (*P* < 0.001, (a)), and severe groups were significantly higher than mild groups (*P* < 0.001, (a)).The ETX levels of severe groups were higher than moderate groups (*P* < 0.05, (b)), moderate groups were higher than mild group (*P* < 0.01, (b)), and severe groups were significantly higher than mild groups (*P* < 0.01, (b)). There was no difference in D-lactate level between each group in active CD patients (*P* > 0.05 for all, (c)).

As was shown in Table 4 and Figure 1, the DAO levels of severe groups were higher than those of moderate groups (*P* < 0.05, Figure 1(a)), and the DAO levels of moderate groups were also higher than those of mild groups (*P* < 0.001, Figure 1(a)). The ETX levels of severe groups were higher than those of moderate groups (*P* < 0.05, Figure 1(b)), and the ETX levels of moderate groups were also higher than those of mild group (*P* < 0.01, Figure 1(b)). In brief, as severity of endoscopy increased, the levels of DAO and ETX increased. There was no difference in D-lactate levels among three groups of active CD patients (Figure 1(c)).

**Figure 2:**
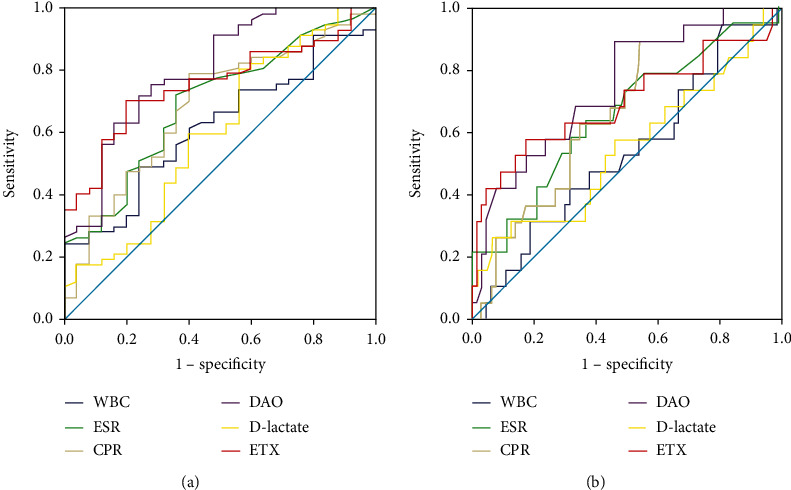
Receiver operator curves (ROCs) of DAO, D-lactate, ETX, and inflammatory markers in detecting IBD severity corresponding to different endoscopic scoring thresholds. The results showed that DAO and ETX are valuable in differentiating CD patients with different endoscopic activity levels. The ROC curve at SES − CD ≤ 6 is shown in (a), and the diagnostic value of DAO is better than ETX. As well as the ROC curve at SES − CD < 16 is shown in (b), and the diagnostic value of DAO is better than ETX. D-lactate, WBC, ESR, and CRP failed to predict CD endoscopy severity.

The DAO and ETX had diagnostic utility in differentiating CD patients with different endoscopic activities (Figure 2 and Table 4). For DAO, the optimum discriminative cutoff threshold for distinguishing endoscopic mild disease was 18.54 u/L, with an AUC of 0.80, *P* < 0.001 (sensitivity 71.93%, specificity 71.93%, PPV 87.2%, NPV 54.3%, and accuracy 71.95%). For ETX, the optimum discriminative cutoff threshold for distinguishing endoscopic mild disease was 10.51 u/L, AUC was 0.76 (*P* < 0.001), sensitivity was 70.18%, specificity was 80.0%, corresponding PPV, NPV, and accuracy were 88.9%, 54.1%, and 73.3%, respectively. Further research has found that the optimum discriminative cutoff of DAO threshold for severe disease in CD (SES − CD ≥ 16) was 18.63 U/L (AUC = 0.75, *P* = 0.001). The test parameters of DAO were as follows: sensitivity 89.47%, specificity 53.79%, PPV 37.0%, NPV 94.4%, and accuracy 62.2%. For ETX, the optimum discriminative cutoff for severe disease was 22.38 U/L (sensitivity 57.89%, specificity 82.54%, PPV 50.0%, NPV 86.7%, and accuracy 76.83%, respectively), and the AUC was 0.70, *P* = 0.010 (SES − CD ≥ 16). The D-lactate, as well as WBC, ESR, or CRP, failed to assess endoscopic severity in CD (*P* > 0.05 for all).

### 3.5. Value of Intestinal Barrier Marker in Predicting Endoscopic Remission in CD

**Figure 3:**
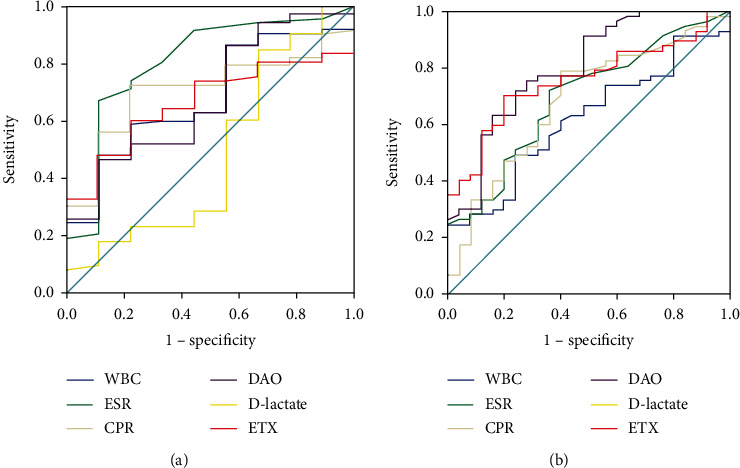
ROC curve of DAO, D-lactate, ETX, and inflammatory markers in predicting CD endoscopic remission. (a) ROC curve at SES − CD ≤ 2. Studies showed that DAO, D-lactate, and ETX values at SES − CD ≤ 2 could not predict the endoscopic remission of CD (*P* > 0.05), nor could WBC, ESR, or CRP. (b) ROC curve at SES − CD ≤ 3. DAO and ETX could predict endoscopic remission of CD with AUC of 0.72 (*P* = 0.008) and 0.67 (*P* = 0.04), respectively, while D-lactate failed to predict remission (*P* > 0.05).

Although we have demonstrated that DAO and ETX were positively correlated with SES-CD, there was no significant difference in level of DAO, D-lactate, and ETX between active and inactive groups in CD patients (*P* > 0.05, respectively; Table 5). The multivariate analyses demonstrated that no optimal threshold could be identified that distinguished active CD from inactive CD, as the NPV of test was not satisfied (Figure 3 and Table 6).

### 3.6. Value of Intestinal Barrier Marker in Predicting Endoscopic Severity in UC

**Figure 4:**
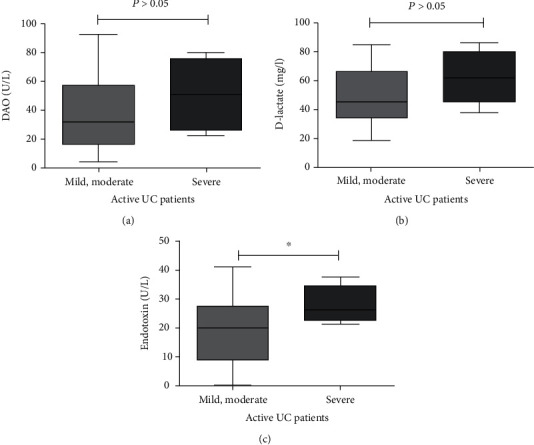
In active UC patients, there was no significant difference in DAO and D-lactate between mild-moderate and severe patients (*P* > 0.05 for both), while ETX levels of severe group were higher than mild-moderate group (*P* < 0.05).

**Figure 5:**
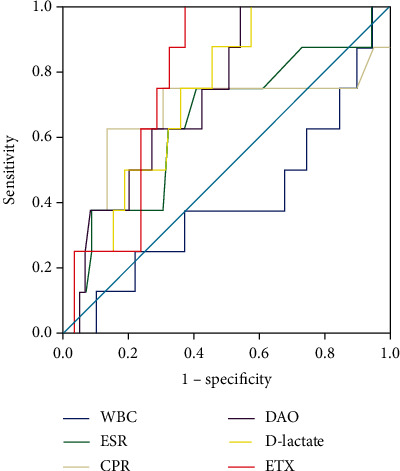
The ROC curves of DAO, D-lactate, ETX, and inflammatory markers for detecting the severity of UC endoscopy showed that the above indicators failed to distinguish the endoscopic severity of UC.

In active UC patients, mild and moderate activity was merged into one group. There was no significant difference in DAO and D-lactate between mild-moderate and severe patients (*P* > 0.05 for both, Figure 4), and only levels of ETX for severe group were higher than mild-moderate group (*P* < 0.05, Figure 4). The result is quite different with CD. ROC curve analysis was shown in Figure 5 and Table 7. No endoscopic disease category was individually distinguishable by DAO, D-lactate, ETX, WBC, ESR, and CRP. Although the levels of ETX of severe group were higher than mild-moderate group, we have not found an optimum discriminative cutoff of ETX to distinguish the severity of endoscopy.

### 3.7. Value of Intestinal Barrier Marker in Predicting Endoscopic Remission in UC

**Figure 6:**
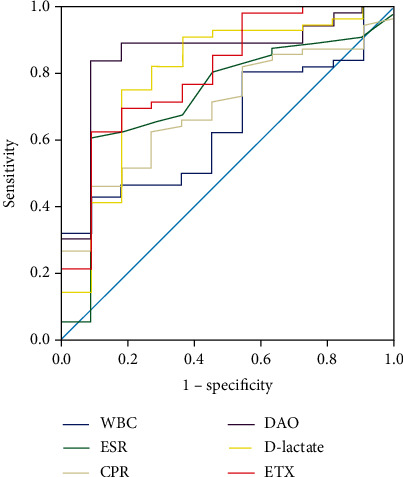
ROC curve of DAO, D-lactate, ETX, and inflammatory markers for UC endoscopic remission (UCEIS ≤ 1). The results showed that DAO, D-lactate, and ETX had diagnostic value in differentiating remission (UCEIS ≤ 1) from activity (UCEIS > 1) of UC patients.

Significant differences were found in DAO, D-lactate, and ETX levels between active and inactive UC patients (*P* = 0.004, *P* = 0.008, and *P* = 0.003; Table 8). On the whole, the levels of DAO, D-lactate, and ETX of the active group were higher than that of the inactive group (UCEIS ≤ 1). ROC analysis shown that DAO, D-lactate, and ETX have a certain diagnostic utility in predicting endoscopic remission (Table 9 and Figure 6). The AUC of DAO for endoscopic disease thresholds of UCEIS ≤ 1 was 0.86, *P* < 0.001. The optimum discriminative cutoff of DAO threshold was 15.97 U/L, predicting endoscopic remission with 83.93% sensitivity and 90.91% specificity; the PPV, NPV, and accuracy of this threshold were 97.9%, 52.6%, and 85.07%. The AUC for D-lactate and ETX for endoscopic disease thresholds of UCEIS ≤ 1 was 0.80, *P* = 0.002 and 0.80, *P* = 0.002, the corresponding cutoff of D-lactate and ETX is 36.53 mg/l and 17.55 U/L. The sensitivity, specificity, PPV, NPV, and accuracy of D-lactate in predicting remission of this thresholds are 75%, 81.82%, 95.5%, 39.1%, and 76.12%. The sensitivity, specificity, PPV, NPV, and accuracy of ETX in predicting remission of this thresholds are 62.5%, 92.91%, 97.2%, 32.3%, and 67.16%.

We further repeated the experiment, and the results showed that if UCEIS = 0 was defined as remission, there was no statistically significant difference in the level of DAO, D-lactate, and ETX of UC patients with active patients (*P* > 0.05). ROC curve analysis also showed that DAO, D-lactate, and ETX could not predict endoscopic UC remission when UCEIS = 0 was defined as remission.

## 4. Discussion

The activity of IBD is closely related to readmission rates and prognosis of patients and leading to significant increases in morbidity and mortality. In patients with inflammatory bowel disease, endoscopy plays a key role in the assessment of disease activity, disease recurrence, treatment response, dysplasia surveillance, and delivery of endoscopic therapy [18]. Although less invasive biomarkers are in development, diagnosis still relies on endoscopy and histological assessment of biopsy specimens [19]. It is quite a difficult task to find an accurate, reliable, and reproducible noninvasive biomarker to replace endoscope. Currently, DAO, D-lactate, and ETX are used to evaluate the function of the intestinal barrier which plays an important role in the development of IBD by assessing intestinal permeability changes, intestinal bacterial migration, and intestinal injury [20]. In this study, we evaluated the correlation between DAO, D-lactate, ETX, and IBD endoscopic score, as well as their value as alternative markers in predicting IBD endoscopic activity and remission. Our results showed that DAO and ETX are positively correlated with SES-CD in CD patients, but D-lactate is not significantly correlated with SES-CD. In UC, DAO, D-lactate, and ETX are also positively correlated with UCEIS. In addition, we found that DAO, D-lactate, and ETX could be used to distinguish endoscopic severity of CD patients to some degree, as well as that DAO, D-lactate, and ETX could be used to distinguish endoscopic remission in UC patients.

Diamine oxidase (DAO) is an enzyme mainly located in villus tip enterocytes of humans and other mammals, which mainly expressed in intestinal mucosal. The level of DAO in peripheral blood is usually very low. The DAO would release into the circulation when the intestinal mucosal cells were damaged, resulting in the increase of DAO in blood. Several previous researches of humans and rats have suggested that its activity reflected the integrity of intestinal mucosa [21], as well as injury and recovery [22, 23]. In humans, D-lactate is mainly produced by metabolism of indigenous bacteria in the gastrointestinal tract. Studies indicated that mammals are not equipped with efficient enzyme systems to metabolize D-lactate [24, 25]. As a consequence, D-lactate tends to be maintained at low levels in healthy individuals. When the intestinal mucosa barrier was injured, D-lactate could be released to the blood through the dysfunctional intestinal barrier, leading to the increase of serum D-lactate. Endotoxin (ETX) is the outer membrane of gram-negative bacteria cell walls of lipopolysaccharide (LPS), usually released after the death of a bacterium, as well as in the metabolic process. Intestinal tract is the body's largest bacterial endotoxin storage, and endotoxin enters the blood circulation with unknown mechanism through the damaged intestinal mucosa barrier. A study of 18 CD patients suggested that the level of serum ETX was elevated during the acute exacerbation [26], and the consequence was confirmed by several other studies [27].

A study indicated that the levels of serum DAO and D-lactate are higher in IBD patients compared with those in healthy groups, and serum DAO and D-lactate could be decreased after treatment [28], suggesting that IBD patients suffer intestinal barrier dysfunction. In this study, DAO and ETX were found to be associated with CD endoscopy score (SES-CD) in patients with CD, and the correlation coefficient was superior to traditional inflammatory markers (WBC, ESR, and CRP). DAO, D-lactate, and ETX were significantly correlated with UCEIS in UC patients, and the correlation coefficient was also superior to traditional inflammatory markers. Our hypothesis was proved that DAO, D-lactate, and ETX showed significant intestinal specificity compared with traditional inflammatory markers (WBC, ESR, and CRP). The verification could explain that why DAO, D-lactate, and ETX were more correlated with IBD endoscopic score than traditional inflammatory markers. At the same time, our results showed that DAO, D-lactate, and ETX were more correlated with UC endoscopic score than CD. However, it is worth noting that our results showed no significant correlation between D-lactate and SES-CD. In addition, due to the limitation of retrospective study, changes in DAO, D-lactate, and ETX levels and their correlation with endoscopic scores after treatment have not been studied, which requires further prospective studies.

In the study of patients with different endoscopic activity levels (severity), our results showed that in patients with CD, DAO level in patients with severe activity is higher than that in patients with moderate activity, and DAO level in patients with moderate activity is also higher than that in patients with mild activity. The same was true for the analysis in EXT. Overall, DAO and ETX levels showed an upward trend with the increase of endoscopic activity. However, there were no statistically significant differences in D-lactate levels among CD patients with mild, moderate, and severe activity, which was consistent with the correlation analysis results, suggesting that CD patients with high levels of DAO and ETX tend to be with more severe endoscopic activities.

Further analysis suggested that DAO and ETX have certain diagnostic value in distinguishing CD patients with different endoscopic activity. ROC curves showed that DAO and ETX could be used to distinguish moderate and severe endoscopic activity. The thresholds for identifying endoscopic moderately active patients with mild activity were 18.54 U/L (sensitivity 71.93% and specificity 71.93%) and 10.51 U/L (sensitivity 70.18% and specificity 80.0%). The best discriminant threshold for identifying DAO thresholds for patients with severe activity was 18.63 U/L (sensitivity 89.47% and specificity 53.79%). For ETX, the threshold for predicting patients with severe activity was 22.38 U/L (sensitivity was 57.89%, and specificity was 82.54%). D-lactate was unable to assess the different activities of endoscopy in the CD, which was consistent with the results of the correlation analysis. Salim and Soderholm [12] believed that intestinal mucosal barrier is severely damaged during acute IBD attack, resulting in structural and functional destruction, and subsequently, increased permeability and tissue damage. A study of 18 CD patients showed elevated levels of ETX during acute episodes in CD patients [26]. Our research confirmed this point, and it also indicated that patients with high levels of DAO and ETX are prone to be associated with more serious endoscopic activities.

Although DAO and ETX are proved to be positively correlated with SES-CD in this study, when SES − CD ≤ 2 was defined as endoscopic remission, the difference between DAO and ETX among patients with activity and remission was not statistically significant. ROC curve analysis also showed that DAO and ETX could not identify remission and activity (*P* > 0.05). There was no statistical difference in D-lactate level between patients with activity and remission, and ROC curve analysis also showed that D-lactate has no diagnostic value in predicting endoscopic remission at SES − CD ≤ 2. Thus, no threshold was found for the best intestinal barrier marker to distinguish CD activity from remission. Pastor Rojo et al. [27] found that the serum endotoxin level increased in IBD patients, although decreased after treatment, ETX did not fully return to normal levels in CD patients. The reason may be related to the recovery degree of intestinal barrier function in patients with remission. Several studies suggested that patients in remission stage are capable to be associated with increased intestinal permeability [29, 30]. Combined with our study, the possible cause is that the intestinal barrier function of CD patients is still not fully recovered, although the intestinal mucosa heals after treatment. Chang et al. [31] found that the degree of intestinal permeability damage is related to the severity of diarrhea, therefore, not only mucosal healing should be included in the final treatment goal of IBD but also the recovery of intestinal barrier function. Some studies showed that CD patients with increased intestinal permeability in remission stage also have an increased risk of disease recurrence [32]. Although DAO and ETX were not proved to be valuable in predicting endoscopic remission of CD, our study demonstrated that further therapy for intestinal barrier was still needed in CD patients at endoscopic remission stage. Continuous treatment is capable to be helpful for reducing CD recurrence and maintaining longer remission period.

However, in UC patients with different endoscopic activity, there was no significant difference in DAO and D-lactate between the mild moderate activity group and the severe group, but the ETX levels of severe group were significantly higher compared with the mild-moderate group. Although high level of ETX tended to be associated with more serious endoscopic activity, we did not detect the best ETX discriminant threshold. Correlation analysis showed that DAO, D-lactate, and ETX were correlated with UCEIS up to a point, but with the increase of endoscopic severity, the levels of DAO, D-lactate, and ETX increased not as obvious as CD. This may be related to the fact that UC is a lesion limited to submucosa, and CD is a transmural lesion of whole mucosa.

Therefore, when distinguishing endoscopic relief from activity, ROC curve analysis showed that DAO, D-lactate, and ETX could predict endoscopic UC relief (UCEIS ≤ 1). There were significant differences in DAO, D-lactate, and ETX levels between UC patients with activity (UCEIS > 1) and remission (UCEIS ≤ 1) (*P* = 0.004, *P* = 0.008, and *P* = 0.003); and DAO, D-lactate, and ETX levels in the active group were significantly higher than those in the remission group. The best prediction boundary of DAO between activity (UCEIS > 1) and remission (UCEIS ≤ 1) was 15.97 U/L, with a sensitivity of 83.93% and specificity of 90.91% for predicting endoscopic remission and an accuracy of 85.07%. The best predictive values of D-lactate and ETX for activity (UCEIS > 1) and remission (UCEIS ≤ 1) were 36.53 mg/l (sensitivity 75% and specificity 81.82%) and 17.55 U/l (sensitivity 62.5% and specificity 92.91%), with an accuracy of 76.12% and 67.16%, respectively. Further repeated analysis was performed at UCEIS = 0 and UCEIS ≤ 2, and the results also fell short of expectations.

Combined with correlation analysis and detection analysis results of different activity levels, although DAO, D-lactate, and ETX could not distinguish UC endoscopic activity level, our study showed that UC patients with low level DAO, D-lactate, and ETX were more inclined to endoscopic remission. Pastor Rojo et al. [27] found that endotoxin levels were reduced in patients with IBD after treatment, but CD patients did not fully return to normal levels. Part of the description showed that compared with CD, UC patients are more likely to achieve recovery of intestinal barrier function during remission, which is subject to further prospective trials. Our research showed that DAO, D-lactate, and ETX have good predictive value of endoscopic remission in UC and have potential application value in clinical.

To sum up, not only endoscopic examination, biomarkers are of great importance to assessing IBD progress. Our study found that DAO, D-lactate, and ETX are significantly correlated with UCEIS score in UC patients, and the correlation coefficient was better than traditional inflammatory markers WBC, ESR, and CRP. We also found that in patients with CD, DAO, and ETX not only showed better correlation with SES-CD than WBC, ESR, and CRP but also could distinguish different endoscopic activities. Due to the influence of sample size and the nature of retrospective study, our study still has some limitations. Further prospective trials are necessary in the future. It is of great significance for clinical practice to develop treatment strategies and predict prognosis.

## Figures and Tables

**Table 1:** Baseline characteristics of patients with CD and UC.

	Total (*n* = 149)	CD (*n* = 82)	UC (*n* = 67)
Median age (years, IQR)	31.5 (24.0-47.3)	28.0 (21.0-33.0)	46.0 (29.0-49.0)
Median disease duration (years, IQR)	3.0 (1.0-4.0)	3.0(1.0-4.0)	3.0 (0.5-4.0)
Gender (%)			
Male	100 (67.11)	56 (68.29)	44 (65.67)
Female	49 (32.89)	26 (31.71)	23 (34.33)
Smoking history (%)			
Yes	10 (6.71)	3 (3.66)	7 (10.45)
No	139 (93.29)	79 (96.34)	60 (89.55)
CD location (%)			
Ileal (L1)	19 (12.67)	19 (22.90)	—
Colonic (L2)	18 (12.00)	18 (21.69)	—
Ileocolonic (L3)	44 (29.53)	44 (53.65)	—
Upper gastrointestinal involvement (L4)	1(0.67)	1(1.22)	—
UC extent (Montreal) (%)			
Proctitis (E1)	9 (6.00)	—	9 (13.43)
Left-sided (E2)	15 (10.00)	—	15 (22.39)
Extensive (E3)	43 (28.67)	—	43 (64.18)
Medication			
None	12 (8.00)	9 (10.84)	3 (4.48)
5-ASA	96 (64.43)	34 (41.46)	62 (92.54)
Steroids	32 (21.33)	14 (16.87)	18 (26.87)
Thiopurine	15 (10)	13 (15.67)	2 (2.99)
Anti-TNF	46 (30.67)	43 (51.80)	3 (4.48)

**Table 2:** Coefficient of correlation between intestinal barrier index, inflammatory markers, and endoscopic scores of disease severity in CD patients.

	Pearson' *r*	*P*
White blood cells	0.146	0.191
Erythrocyte sedimentation rate	0.346	0.0015
C reactive protein	0.250	0.023
Diamine oxidase	0.532	<0.0001
D-lactate	0.167	0.133
Endotoxin	0.468	<0.0001

**Table 3:** Coefficient of correlation between intestinal barrier index, inflammatory markers, and endoscopic scores of disease severity in UC patients.

	Pearson' *r*	*P*
White blood cells	0.151	0.222
Erythrocyte sedimentation rate	0.285	0.021
C reactive protein	0.334	0.006
Diamine oxidase	0.600	<0.0001
D-lactate	0.407	0.0006
Endotoxin	0.505	<0.0001

**Table 4:** ROC analysis results of intestinal barrier markers and traditional inflammatory markers at different CD endoscopy activity thresholds.

	SES-CD	
	≤6	<16
DAO	0.80 (0.69-0.90)	0.75 (0.62-0.87)
*P*	<0.001∗∗∗	0.001∗∗
D-lactate	0.60 (0.46-0.74)	0.55 (0.39-0.71)
*P*	0.15	0.492
ETX	0.76 (0.65-0.86)	0.70 (0.54-0.85)
*P*	<0.001∗∗∗	0.010∗
WBC	0.62 (0.49-0.74)	0.53 (0.39-0.68)
*P*	0.094	0.656
ESR	0.69 (0.57-0.81)	0.66 (0.51-0.80)
*P*	0.005∗∗	0.041∗
CRP	0.69 (0.56-0.81)	0.69 (0.55-0.80)
*P*	0.008∗∗	0.020∗

AUC: 95% confidence interval.

**Table 5:** Intestinal barrier markers and inflammatory markers in remission and active CD patients.

	Remission (SES − CD ≤ 2)	Active (SES − CD > 2)	*P*
DAO (U/L)	16.06 ± 12.46	28.63 ± 20.35	0.075
D-lactate (mg/l)	37.78 ± 22.94	37.82 ± 19.84	0.997
ETX (U/L)	9.37 ± 5.51	16.46 ± 13.05	0.113
WBC (10^9^/L)	5.21 ± 1.48	6.62 ± 2.55	0.109
ESR (mm/h)	9.67 ± 14.92	26.39 ± 26.34	0.066
CRP (mg/L)	9.87 ± 12.41	28.55 ± 30.17	0.071

**Table 6:** ROC analysis of intestinal barrier markers and traditional inflammatory markers predicting endoscopic remission in CD.

	SES-CD	
	≤2	≤3
DAO	0.68 (0.51-0.86)	0.72 (0.58-0.86)
*P*	0.074	0.008∗∗
D-lactate	0.49 (0.26-0.71)	0.52 (0.34-0.71)
*P*	0.894	0.77
ETX	0.67 (0.53-0.80)	0.67 (0.55-0.80)
*P*	0.106	0.04∗
WBC	0.67 (0.52-0.83)	0.71 (0.59-0.84)
*P*	0.091	0.010∗
ESR	0.81 (0.65-0.97)	0.70 (0.56-0.84)
*P*	0.003∗∗	0.015∗
CRP	0.71 (0.57-0.85)	0.76 (0.64-0.88)
*P*	0.042∗	0.002∗∗

AUC: 95% confidence interval.

**Table 7:** ROC analysis results of intestinal barrier markers and traditional inflammatory markers at different UC endoscopy activity thresholds.

	UCEIS ≤ 6	*P*
DAO	0.73 (0.58-0.89)	0.034∗
D-lactate	0.74 (0.59-0.89)	0.029∗
ETX	0.78 (0.66-0.90)	0.011∗
WBC	0.40 (0.18-0.62)	0.353
ESR	0.64 (0.41-0.86)	0.216
CRP	0.67 (0.41-0.93)	0.124

AUC: 95% confidence interval.

**Table 8:** Intestinal barrier markers and inflammatory markers in remission and active UC patients.

	Remission (UCEIS ≤ 1)	Active (UCEIS > 1)	*P*
DAO (U/L)	13.52 ± 4.29	38.92 ± 24.61	0.004∗∗
D-lactate (mg/l)	31.41 ± 5.62	51.22 ± 19.78	0.008∗∗
ETX (U/L)	8.47 ± 2.68	20.67 ± 10.73	0.003∗∗
WBC (10^9^/L)	5.74 ± 0.43	6.99 ± 2.44	0.107
ESR (mm/h)	9.73 ± 4.77	19.38 ± 18.57	0.100
CRP (mg/L)	4.27 ± 1.55	23.85 ± 41.18	0.117

**Table 9:** ROC analysis of intestinal barrier markers and traditional inflammatory markers predicting endoscopic remission in UC.

	UCEIS≤1	*P* value
DAO	0.86 (0.74-0.98)	<0.001∗∗∗
D-lactate	0.80 (0.63-0.96)	0.002∗∗
ETX	0.80 (0.66-0.95)	0.002∗∗
WBC	0.64 (0.49-0.80)	0.136
ESR	0.72 (0.57-0.89)	0.018∗
CRP	0.70 (0.55-0.84)	0.041∗

AUC: 95% confidence interval.

## Data Availability

All data used during the study appear in the submitted article.
